# Patients’ acceptability of self-selected digital health services to support diet and exercise among people with complex chronic conditions: Mixed methods study

**DOI:** 10.1177/20552076241245278

**Published:** 2024-06-07

**Authors:** Amandine Barnett, Soraia de Camargo Catapan, Dev K Jegatheesan, Marguerite M Conley, Shelley E Keating, Hannah L Mayr, Lindsey Webb, Riley C C Brown, Jeff S Coombes, Graeme A Macdonald, Nicole M Isbel, Nicola W Burton, Katrina L Campbell, Ingrid J Hickman, Jaimon T Kelly

**Affiliations:** 1 Centre for Online Health, 1974 The University of Queensland, Brisbane, QLD, Australia; 2 Centre for Health Services Research, 1974 The University of Queensland, Brisbane, QLD, Australia; 3 Faculty of Medicine, 1974 The University of Queensland, Brisbane, QLD, Australia; 4 Department of Nephrology, 1966 Princess Alexandra Hospital, Brisbane, QLD, Australia; 5 Department of Nutrition and Dietetics, 1966 Princess Alexandra Hospital, Brisbane, QLD, Australia; 6 School of Human Movement and Nutrition Sciences, 1974 The University of Queensland, Brisbane, QLD, Australia; 7 Centre for Research on Exercise, Physical Activity & Health, 1974 The University of Queensland, Brisbane, QLD, Australia; 8 Centre for Functioning and Health Research, 157827 Metro South Hospital and Health Service, Brisbane, QLD, Australia; 9 Translational Research Institute, Brisbane, QLD, Australia; 10 Department of Gastroenterology and Hepatology, 1966 Princess Alexandra Hospital, Brisbane, QLD, Australia; 11 School of Applied Psychology, 5723 Griffith University, Brisbane, QLD, Australia; 12 Centre for Mental Health, 5723 Griffith University Mount Gravatt, Brisbane, QLD, Australia; 13 Menzies Health Institute Queensland, 5723 Griffith University, Nathan, QLD, Australia; 14 Healthcare Excellence and Innovation, Metro North Hospital and Health Service, Brisbane QLD, Australia; 15 ULTRA Team, The 558219 University of Queensland Clinical Trials Capability, Herston, Brisbane, Australia

**Keywords:** Digital health, telehealth, mHealth, eHealth, chronic disease, kidney disease, liver disease, nutrition, exercise, health service

## Abstract

**Objective:**

The acceptability of being offered a choice from a suite of digital health service options to support optimal diet and exercise behaviors in adults with complex chronic conditions was evaluated. This study sought to understand many areas of acceptability including satisfaction, ease of use, usefulness and user appropriateness and perceived effectiveness.

**Methods:**

This mixed-methods study was embedded within a randomized-controlled feasibility trial providing digital health services managing diet and exercise for adults from specialist kidney and liver disease clinics. Post study surveys and semistructured interviews were used to determine patients’ acceptability of the trial interventions. Quantitative (surveys) and qualitative (surveys and interviews) results were merged using integrative analysis and mapped to each construct of the modified version of the Theoretical Framework of Acceptability.

**Results:**

Seventeen interviews (intervention group) and 50 surveys (*n* = 24 intervention, *n* = 26 comparator) completed from a possible 67 participants were analyzed. In the intervention group, the survey results revealed high areas of acceptability for the digital health services including overall support received, ease of use, timely advice and feeling safe. The interviews also revealed high areas of acceptability including convenience, ability to adopt healthier behaviors and having regular interactions with health professionals. However, the interviews also revealed lower areas of acceptability as a result of absence of individualization, low digital literacy, and limitations from life circumstances.

**Conclusions:**

Recipients of digital health services that supported diet and exercise interventions found these useful, effective, and safe. Individualized care, technical support and patient confidence remain important to improve the acceptability of digital health service interventions.

## Introduction

Chronic conditions are the leading cause of death and disability worldwide, accounting for seven out of 10 premature deaths globally^1^ with an estimated 100 million additional quality-adjusted life years lost in 2019.^2^ Healthy lifestyle behaviors that include a high-quality diet and regular physical activity are a first-line treatment strategy in this population.^3–5^

Patient access to diet and exercise support across Australian public health services are often fragmented and under resourced,^6,7^ relative to the increasing demand complex chronic conditions are placing on the system. Several barriers exist to receiving high quality care, including services being siloed to disease specialities with an increased risk that patients with multimorbidities receiving either repetitious or conflicting diet and exercise advice from a variety of practitioners.^8^ At the organizational-level, often tertiary health services are faced with high patient cancelation rates,^9^ which continues to be a significant resource burden in outpatient settings. Some of the reasons for lack of attendance relate to overcrowding, long wait times, lack of continuity, infrequent interactions with healthcare professionals, being too unwell to attend, impact on work commitments, travel, and associated costs.^10^ There is also little shared decision making with regards to when, where and how patients receive healthcare.^11^ Therefore, there is a need to focus on innovative health services which can improve the patient's experience and result in more reliable attendance.

Digital health services are well suited to the management of chronic conditions as they can support assessment and self-monitoring, facilitate virtual repeated contacts with health professionals and offer on-demand access to digital resources.^12^ Digital health can involve a wide range of service-provision modalities, including (but not limited to), mobile health applications, video consultations and wearable devices to track behavior.^13^ Recent reviews investigating the use of digital health in chronic condition populations have demonstrated improvements in diet quality^14,15^ and physical activity.^16,17^ This may be related to improvements in self-efficacy and self-management of lifestyle-related behaviors such as diet and exercise.^18^ In addition, there is evidence that digital health lifestyle interventions are feasible for both patient use and service provision for complex chronic conditions.^19–22^ However, these previous interventions are typically highly prescriptive with limited opportunity for patient choice in the type of digital health services used and amount of engagement with these services.^19–22^

Integrating patient choice within an intervention could be key to individualizing healthcare and improving clinical outcomes.^23,24^ Such interventions require rigorous evaluation involving evidence-based approaches. The acceptability of various interventions, including those in digital health, has been evaluated using the Theoretical Framework of Acceptability (TFA),^25–29^ which consists of seven component constructs.^30^ It provides a robust framework for evaluating the acceptability which is important as digital health interventions can be highly complex involving multiple components, interactions, and outcomes. Therefore, the aim of this study was to evaluate the acceptability of being offered a choice from a suite of digital health service options to support optimal diet and exercise behaviors in adults with complex chronic conditions by utilizing the constructs of the TFA.

## Mixed Method

### Study design and setting

This convergent parallel mixed-methods study^31^ was embedded in a single-center 26-week feasibility randomized controlled trial.^32,33^ The convergent parallel mixed-methods study design, which allows complementary data to be analyzed separately and then integrated together,^31^ helped to address the complexity of the research aim. The Utilizing technology for Diet & Exercise Change In complex chronic conditions across Diverse Environments (U-DECIDE) trial was conducted from October 2020 to April 2022, to test the feasibility and acceptability of a digital health diet and exercise intervention. The current study is a component of the U-DECIDE randomized controlled trial and details of this prospectively planned acceptability mixed methods analysis are available via the Australia and New Zealand Clinical Trial Registry: ACTRN12620001282976. https://www.anzctr.org.au/Trial/Registration/TrialReview.aspx?id = 378337.

Participants were provided written and/or verbal instructions relative to the digital health materials they used or had access to at the baseline data collection session. All participants provided written informed consent prior to the study initiation. The study was conducted in an Australian public tertiary hospital in Brisbane (Princess Alexandra Hospital). This current study has been reported using the Standards for Reporting Qualitative Research,^3^ Consolidated Criteria for Reporting Qualitative Research,^35^ and the Good Reporting of A Mixed Methods Study checklist^36^ (Supplementary Material 1-3). Ethics approval for this study was obtained from the Human Research Ethics Committees of Metro South Health (HREC/2019/QMS/58285) and the University of Queensland (2020000127).

### Context

The U-DECIDE study involved a suite of patient selfselected digital health services to support diet and exercise behaviors.^32,33^ In brief, all intervention and comparator participants had an initial appointment with a dietitian and were provided with usual medical and specialist care. The comparator group continued with standard care which involved patient-centered individualized dietary reviews (either face-to-face or telephone) at a frequency deemed appropriate by the treating clinicians. There was no routine access to exercise specialists in the comparator group.

After the initial dietetic appointment, the intervention group received all subsequent dietary education that aligned with heart healthy guidelines^37^ and exercise interventions that aligned with the Australian physical activity guidelines^38^ and follow-up reviews via digital health services. All intervention group participants received:

- Text messages about healthy eating and physical activity underpinned by behavior change techniques including goal setting, social support, information on health consequences and problem solving. These were semi-personalized as participants could select their desired text message frequency (at least once and up to three times per week).

Additionally, they had optional (self-selected) access to:

- Nutrition mobile application (app) (Sophus Nutrition Pty Ltd, Australia) with mobile and web access, which was available throughout the entire study.
- Exercise app (PhysiTrack^®^ Ltd, London/UK) with mobile and web access, which was available throughout the entire study.
- Group-based 45 minute monthly dietetic consultations, via videoconference for five months.
- Group-based 60 minute weekly supervised exercise training sessions, via videoconference for five months.

At the start of the intervention period, the intervention group selected their desired text message frequency and which optional digital health services related to diet and exercise they wished to access.

### Participants and recruitment

A detailed description of the participant selection and recruitment for the U-DECIDE trial is reported elsewhere.^32^ Adults under the outpatient care of specialist kidney and liver disease and kidney/liver transplant clinics were recruited. They were eligible to participate if they had at least one feature of metabolic syndrome^39^ and had been referred to a dietitian to improve diet quality, had access to a mobile device or computer with internet access, and were considered suitable to participate by both a medical and an exercise professional. People who were diagnosed as malnourished, were non-English speaking, had a life expectancy of less than six months, were under 18 or over 80 years of age, were pregnant or breastfeeding were excluded. Computer-generated randomization occurred via REDcap^40^ electronic data capture tool hosted by The University of Queensland, with stratification of groups by referral speciality (kidney, liver and transplant).

All participants who completed the U-DECIDE trial were invited by email by the research project officer to complete an online feedback survey. A sub-sample of the intervention group of the U-DECIDE trial were purposively sampled based on their health condition (chronic liver disease/transplant and chronic kidney disease [CKD]/transplant) and self-selected digital health use (nutrition app, exercise app, diet and/or exercise video consultations). This group were contacted by phone call and invited by the researcher project officer to provide further in-depth feedback regarding their experience with the study via an individual interview. The participants did not have any knowledge of the interviewer.

### Data collection

This study used participants’ characteristics data from the U-DECIDE trial and collected data on the acceptability of digital health services they utilized using online surveys and interviews. Data were collected between April 2021 and April 2022. Study codes were used to deidentify data and ensure anonymity for participants.

### Participant characteristics

A research project officer with an allied health professional background collected information on the assigned specialist clinic, age and metabolic features of the participants via the hospital's electronic medical records. Other demographic information including gender, ethnicity and education level were self-reported within an online survey via REDcap^40^ prior to U-DECIDE baseline assessment.

### Online surveys

A link to the online feedback survey managed via REDcap, was sent to all U-DECIDE intervention and comparator group participants via email prior to their final assessment visit (six months after baseline collection) (Supplemental Material 4, Table S4-1). The survey assessed the acceptability of care with seven survey items asked of both groups. Questions were designed for both groups to assess perceptions on overall support received, as well as the perceived effectiveness of their respective interventions for improving motivation, understanding and confidence related to diet and exercise. The intervention group received additional questions related to the text messages and each of the digital health service options (nutrition app, exercise app, diet and/or exercise video consultations), with questions corresponding to their individual selection, up to an additional 32 items regarding satisfaction, ease of use, usefulness, user appropriateness, perceived effectiveness, and influence on behavior. Questions were developed and piloted with a diverse clinical and research team from the fields of exercise, nutrition, metabolic medicine, eHealth, and psychology. Participant responses were either a 5- or 6-point Likert scale, yes or no, or short answer and open-ended written responses. All survey data was securely stored and retrieved from REDcap for further download and analysis.

### Interviews

Individual semistructured interviews were conducted by the primary author (AB) who had no role in providing services to the intervention and comparator groups. The primary author had previous experience conducting qualitative research. Interviews were scheduled for approximately 30 minutes either in person at the final assessment visit at the clinic or via a telephone call at the convenience of the participant. Semi-structured interview questions (Supplementary Material 4, Table S4-2) and prompts were developed by the same diverse clinical and research team as the questionnaire, to gain a deeper understanding on the acceptability of digital health services. Adjustments were made to the interview schedule after the first five interviews to improve the clarity of questions. These were audio-recorded in a private setting (clinic room or telephone) with only the researcher and participant present, transcribed verbatim online using Microsoft Azure Cognitive Services^41^ and checked for accuracy against the recording prior to analysis.

### Data analysis

#### Quantitative data

In order to identify significant demographic differences between participants who were included in the current study and those who were not, data were analyzed with descriptive statistics using SPSS (IBM Version 29.0). Shapiro-Wilks test was used to assess the normality of these data. Categorical data was analyzed using the Fisher Exact or Pearson Chi-square nonparametric tests to compare those in the study who completed and those who did not complete the survey.

The same tests were used to identify significant differences between the comparator and intervention groups to understand how their respective interventions impacted their understanding of diet/exercise; their confidence to choose healthy meal and snack options/suitable exercise options; and their motivation to eat healthily and exercise regularly. The survey results were then reported against the seven constructs of the TFA^30^ and the additional construct of safety as proposed by McDonald et al.^42^ The TFA consists of seven constructs namely, Affective Attitude (feelings), Burden (perceived effort), Ethicality (fit with values), Intervention Coherence (comprehensibility), Opportunity Costs (what is given up), Perceived Effectiveness (likelihood of benefit), and Self-Efficacy (confidence to do what is required). An additional construct “perceived safety and risk” has also been added to the original TFA.^42^ Safety can impact other areas of health including lifestyle management particularly when it is delivered remotely via digital health and involves self-management of behaviors.^43^

#### Qualitative data

Individual semistructured interviews were thematically analyzed using an interpretivist approach.^44^ The primary author (AB) familiarized themself with the data and deductively assigned codes to sections of the transcripts that were deemed relevant to the research aim and objectives using NVivo software (Version 12 Pro, QSR International Pty Ltd). Attempts to identify in-vivo codes^45^ were made by highlighting words and phrases that commonly appeared in the transcripts. A subsample of transcripts (*n* = 4) were coded by a second author (SC). To establish validity by triangulation, the two authors (AB and SC) crossed checked and debriefed on code discrepancies. Subsequently, codes that shared similar meanings were categorized into themes. Themes were inductively classified under the modified eight constructs of the TFA for this study. Consensus based discussions were held between authors (AB, SC, JK, and IH) to refine subthemes. Analytical memos were taken, and transcripts were referred to throughout the data analysis process to ensure trustworthiness. Example extracts were selected to represent the various perspectives that emerged from the data, with minor grammatical changes to improve readability. Thematic analysis was also used to analyze the open-ended survey data which was conducted by the primary author (AB) with input received by the other authors. The sample size was informed by information power (as the amount of information for the sample increases, the number of participants required for the sample decreases)^46^ in which recruitment ceased at 17 participants. The research team agreed that there was sufficient information from this sample of 17 participants to identify key themes and to address the TFA.

### Integration and presentation of results

Data integration occurred at the analysis and interpretation phase of the mixed methods design, with several authors (AB, JK, IH, NB, and SC) involved in the process. Quantitative (surveys) and qualitative (surveys and interviews) results were merged using integrative analysis and mapped to each construct of the modified version of the TFA. The themes from the interviews have been italicized in the main text. Definitions for each TFA construct were applied in context with the current study and presented in Table 2. Agree/strongly agree and disagree/strongly disagree Likert scale responses were combined to form two categories of agree and disagree, respectively. This was also done for the Likert scale results dealing with satisfaction, ease of use, helpfulness, and effectiveness. The midcategory options remained unchanged.

## Results

Seventy-five percent (*n* = 50/67) of invited participants submitted survey responses and of the 17 who were invited to interview to provide further feedback, 100% agreed. Participants who did not submit survey responses were either not responsive (*n* = 12) or dropped out of the main U-DECIDE trial (*n* = 5). Mean interview duration was 25 ± 11 minutes. There were no statistical differences in characteristics of study participants who did or did not complete a survey. Participant characteristics are summarized in Table 1. All survey results are further detailed in Supplemental Material 5 and the applied definition for each TFA construct is presented along with the survey components and themes assessed for each construct in Table 2.

**Table 1.** Participant characteristics. Data are median [IQR] or *n* (%).

Characteristic	Completed survey (*n* = 50)	Interviewed (*n* = 17)
Years of age	53 [40–62]	55 (38–64)
Male	28 (56)	10 (59)
Health condition		
Chronic kidney disease	20 (40)	6 (35)
Liver disease	5 (10)	2 (12)
Kidney transplant	16 (32)	8 (47)
Liver transplant	9 (18)	1 (6)
Presence of metabolic syndrome features		
Hypertension	30 (60)	10 (59)
Central obesity	31 (62)	12 (70)
Hyperglycemia	13 (26)	6 (35)
Dyslipidemia	19 (38)	5 (29)
Ethnicity		
European Australian	41 (82)	15 (88)
Indigenous	3 (6)	1 (6)
Asian	4 (8)	3 (18)
Other	6 (12)	0 (0)
Education		
High school at least grade 10	4 (8)	1 (6)
High school grade 10	6 (12)	3 (18)
High school grade 12	7 (14)	1 (6)
Vocational college	18 (36)	7 (41)
University	15 (30)	5 (29)
Group allocation		
Intervention	24 (48)	17 (100)
Comparator	26 (52)	N/A

Abbreviations: IQR, Inter Quartile Range; n, sample number of participants; N/A, Not Applicable.

**Table 2.** The modified eight construct Theoretical Framework of Acceptability^30,42^ with definitions applied to the current study.

Acceptability Construct	Applied definition of construct in the current study	Related results: Survey assessment and interview themes
Affective attitude	How participants feel about the digital health diet and exercise service/s	**Survey:** satisfaction, recommending to others **Interviews:** useful for others with limited knowledge and skills; value of peer and professional support
Burden	The perceived effort required to engage with the digital health diet and exercise service/s	**Survey:** ease of use, technical issues **Interviews:** varied levels of digital health engagement
Ethicality	The extent to which receiving the digital health diet and exercise service/s aligns with participants’ values	**Survey:** sharing resources with others, relevance **Interviews:** convenience of when I can engage in digital health; comparing digital health with previous experiences of in-person care; additional digital health features desired
Intervention coherence	The extent to which participants understand the digital health diet and exercise service/s and its recommendations and how it works.	**Survey:** uncertainty with correct use of digital health **Interviews:** understanding purpose of digital health; lacking digital competence
Opportunity costs	The extent to which benefits, profits or values must be given to engage with the digital health diet and exercise service/s and follow requirements	**Survey:** timing of when digital health was provided **Interviews:** life circumstances impacting capacity to make behavior change
Perceived effectiveness	The extent to which the digital health diet and exercise service/s are likely to promote positive change: engagement with materials, perceived helpfulness,	**Survey:** improvements to motivation; understanding and confidence, changes to behavior; helpfulness **Interviews:** helped to adopt healthier behaviors; useful for others with limited knowledge and skills; reaffirmed what I knew
Self-efficacy	The confidence to perform the behavior(s) required	**Survey:** capability to exercise. **Interviews:** lacking digital competence, awareness of exercise capabilities; desire for more tailored and practical approaches
**Additional construct**		
Perceived Safety	Factors contributing to personal protection or risk during participation in a digital health and exercise service/s and resources	**Survey:** feeling safe **Interviews:** professional support to adapt to current health needs

### Affective attitude (satisfaction)

**Figure 1.**
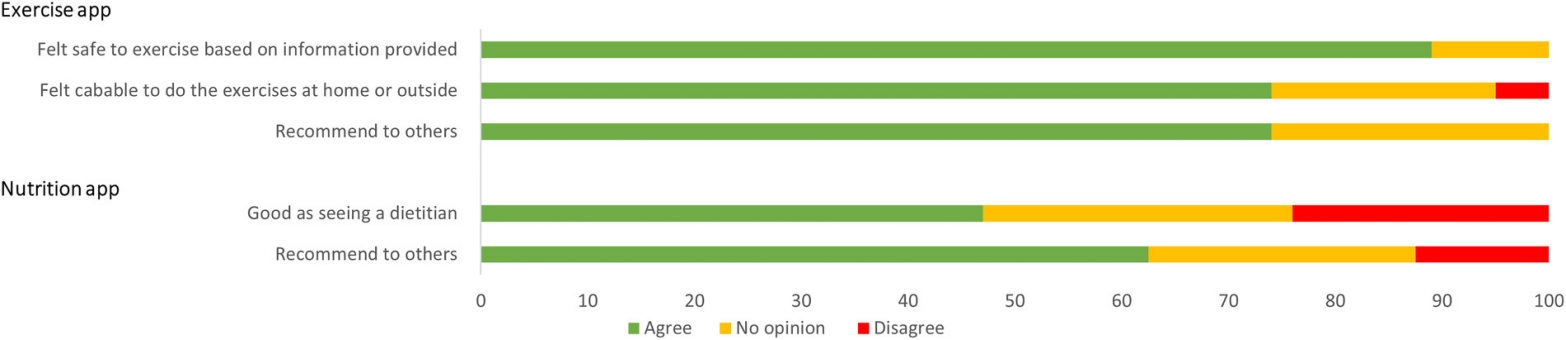
A bar graph showing how they feel about the exercise app (*n* = 22) and the nutrition app (*n* = 21) for those who selected these digital health service options in the intervention group. Agree refers to agree and strongly agree combined; and disagree refers to disagree and strongly disagree combined. The top half of the graph relates to the exercise app and bottom half relates to the nutrition app. Please note that the participants who selected these digital health service options but did not use the app were not included in this graph.

The survey results showed 83% (*n* = 20/24) of participants in the intervention group were satisfied with the overall support received, which was higher, but not statistically different from the comparator group (58% *n* = 15/26, *p* = .07). Just less than three-quarters of intervention participants (70%, *n* = 14/20) were satisfied with the exercise video consultations and just over half (55%, *n* = 11/20) were satisfied with the diet video consultations. Eighty-three percent (*n* = 20/24) of intervention participants agreed that digital health was something that they could see being offered long term to other hospital outpatient clinics. Forty-eight percent (*n* = 10/21) of participants agreed that they would recommend the nutrition app to others and 64% (*n* = 14/22) agreed that they would recommend the exercise app to others (Figure 1).

Two interview themes reflected the affective attitudes construct. Participants reported that digital health services may be *useful for others with limited knowledge and skills*. For example, they felt the recipe feature in the nutrition app would be more useful to others than it was to themselves: “Maybe for somebody (who) doesn’t cook … (it is) more useful” (63-years male, CKD).

Interview data indicated a theme that there were mixed attitudes toward the value of peer and professional support. Some participants had positive experiences in which they shared ideas and were inspired by discussion within their diet video consultations: “It was actually really good to have another study person because I picked up stuff from them” (47-years male, CKD). However, a minority of participants who were not exposed to a group setting had opposing views and were concerned about relevance of the group discussions: “The thought of just sitting online chatting to a load of old men about their bacon sandwiches just was like … I don't think I'll get much benefit from it, if you know what I mean” (40-years female, CKD). For some participants who had selected the exercise video consultations, scheduling a time held them accountable to attend sessions where they knew other participants and health professionals were involved: “I think with an exercise physiologist it was good because I knew I had to get up in the morning and do it … if I'm accountable, I'll do it” (65-years male, kidney transplant).

### Burden (ease of use)

**Figure 2.**
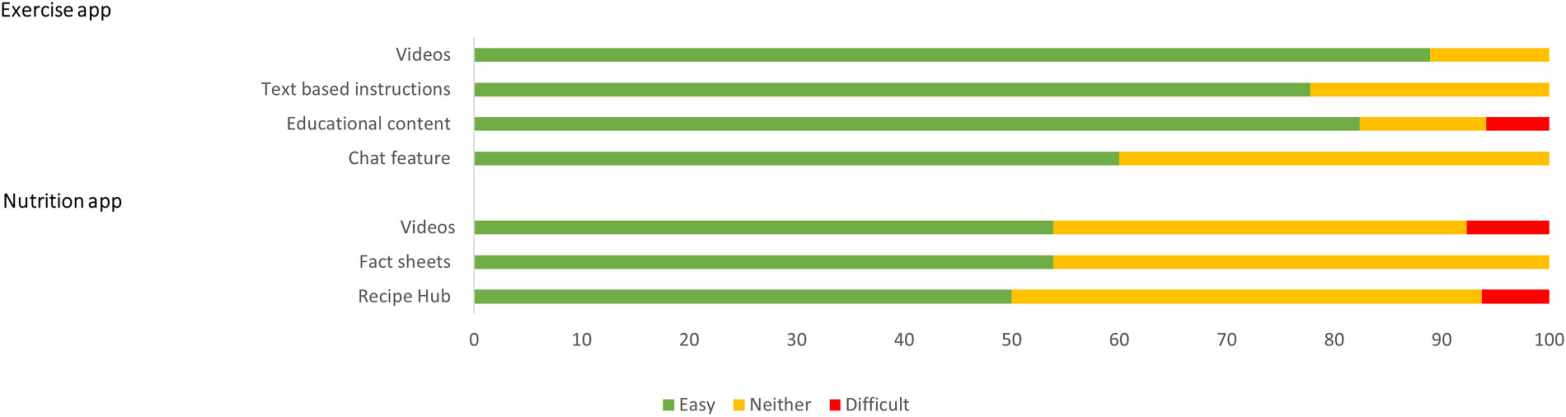
A bar graph showing perceived ease of use of the features embedded in the exercise app (*n* = 22) and nutrition app (n = 21) for those who selected these digital health service options in the intervention group. Easy refers to easy and very easy combined; and difficult refers to difficult and very difficult combined. The top half of the graph relates to the exercise app and bottom half relates to the nutrition app. Please note that the participants who selected these digital health service options but did not use the app were not included in this graph.

Survey and interview results indicated mixed degree of burden. The survey revealed that technical problems were encountered by approximately one in five participants using the digital health services (21%, *n* = 5/24). Of those who used the nutrition app, 33% (*n* = 7/21) found it easy to use. However, of those who used the exercise app, 73% (*n* = 16/22) found that the exercise video demonstrations were easy to use and 64% (n = 14/22) found that the educational content was easy to use. Participants also indicated the ease of use with the recipes and educational videos for the nutrition app; and the text-based instructions of exercises and built-in chat features with the exercise physiologist for the exercise app (Figure 2).

Varied levels of digital health engagement was a theme that emerged from the interviews related to the burden construct. Some participants reported that their engagement with the exercise app ranged from weekly to monthly use. Most participants who chose to use the nutrition app reported briefly looking at the resources at the beginning of the study: “Yeah, flicked through a few recipes, so not much (use)” (55-years female, CKD). Some indicated that they read the text messages straight away, while others read them later or did not read them at all. Several participants admitted to forgetting the digital health services they selected including the video consultations and the apps: “Sorry, I don’t remember using it” (22-years male, liver disease).

### Ethicality (matching core values)

The survey and interview results show a mixed degree to which participants thought the intervention matched their values. Eighty-eight percent (*n* = 21/24) of participants agreed that the exercise information provided digitally was relevant and 58% (*n* = 14/24) agreed that the diet information accessed digitally was relevant. The survey results indicated that the diet and exercise information received was shared with family and friends by 38% (*n* = 9/24) of participants. However, in the survey’s open-ended response, a minority of participants declared that the advice received did not seem relevant to their health condition. The survey results indicated that diet and exercise information received was shared with family and friends by 38% (*n* = 9/24) of participants.

There were three themes related to participant values. *Convenience of when I can engage in digital health* was an interview theme. Participants valued being able to reschedule video consultation times: “having that option to reschedule made it a lot better, instead of say I'll just see you next week” (65-years male, kidney transplant). They also valued accessing digital health at any time and place that suited them: “With the (exercise app) … I can do it anytime” (55-years female, CKD).

*Comparing digital health with previous experiences of in-person care* was another interview theme that reflected participant values. Some participants felt that diet and exercise video consultations were similar or better than in-person care they had experienced previously outside of the research context, describing it as feeling more personal: “I think it's actually better online because I feel like she took her time more. Whereas over here (outpatient clinic) sometimes you know you get disrupted by someone coming in and things. Or (it) … felt like sometimes it's a tiny bit rushed” (27-years female, kidney transplant). Conversely a small number of participants preferred in-person care as they perceive it to be more motivating particularly when it came to exercise: “it just doesn't really create the nicest atmosphere to be like doing it with the screen. It is not as motivating, just like looking at my phone” (26-years female, kidney transplant).

The interview theme of *additional digital health features desired* indicated that extra components could improve the value of the digital health services, specifically in relation to the apps. This included reminders to attend the video consultations or notifications to prompt use of the apps they selected: “So that it would prompt me to want to go back” (26-years female, kidney transplant). Participants also suggested having a grocery shopping list linked to the recipe options in the nutrition app and an all-inclusive and integrated nutrition and exercise app.

### Intervention coherence (understanding of requirements)

In the survey’s open-ended response, participants also described that digital health was challenging and expressed uncertainty about whether they were using it correctly.

The interviews demonstrated varying comprehension toward the digital health services. The theme *understanding purpose of digital health* indicated there was intervention coherence. Some participants indicated that the video features in the nutrition and exercise app were helpful, especially for those who were not confident readers: “Yes, it (videos) was more helpful to someone like me than somebody that could read the whole thing. Yes, because I could then understand it” (35-years female, kidney transplant). Several participants recognized that the information received between the digital health services aligned: “Well it matched up …. what he (the exercise physiologist) was doing and what the (exercise app) does is very much inline which I thought was really important because if they give you exercises that you don’t know anything about even though you watch the video, you can overdo it quite easily (65-years male, CKD).

However, the theme *lacking digital competence* indicated challenges for comprehensibility. Some participants were challenged with opening the nutrition app or connecting to video consults and were unsure how to troubleshoot the issue “I wasn’t sure what I was supposed to do (when connecting to the video consultations)” (22-years male, liver disease).

### Opportunity costs (benefits, profits, and values given to engage)

The survey data revealed that 71% (*n* = 17/24) frequently read the text messages and 79% (*n* = 19/24) thought they were timely.

The interviews revealed that common “costs” of using digital health services were competing with life demands and time. The theme *life circumstances impacting capacity to make behavior change* highlighted a common opportunity cost. Participants described challenges in dealing with symptoms, treatment requirements and competing priorities: “I go through patches where I feel unwell to go and do anything like walk, even walking it knocks me around … the days I feel good, I've got many things to do … rather than just exercise for the sake of exercise” (64-years male, CKD). Their condition also made it challenging to be certain of their progress with lifestyle behavior change such as weight loss: “Having issues with fluid, meant that the weight kept on going up and then it was all over the shop and it was hard to gauge whether [I] was actually losing weight because of diet or whether I was losing it because I was on diuretics” (65-years male, kidney transplant). For some, work and family commitments constrained behavior change: “I guess you could say like work and you know commitments with the kids, that's with school and sport after school and it was just hard to sort of incorporate what I was trying to do with the rest of the family” (48-years male, liver disease).

### Perceived effectiveness (positive impact on motivation and behavior change)

**Figure 3.**
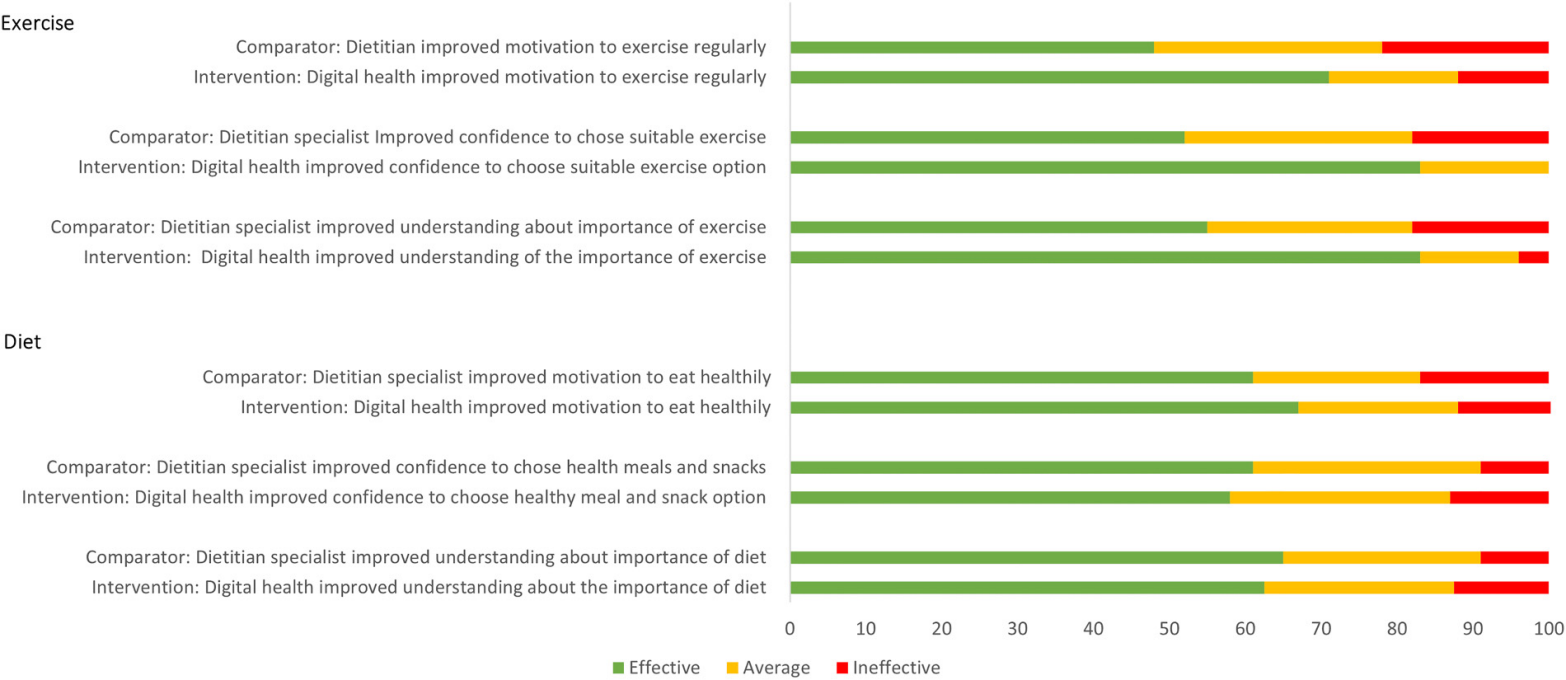
A bar graph showing the perceived effectiveness of the comparator (*n* = 23) or intervention (*n* = 24) at improving motivation, confidence and understanding of diet and exercise for both groups. Effective refers to effective and very effective combined; and ineffective refers to ineffective and very infective combined. The top half of the graph relates to exercise and bottom half relates to diet.

There were similar perceptions of the effectiveness of digital health and usual care services to improve motivation to eat healthily, 67% (*n *= 16/24) in the intervention group versus 61% (*n *= 14/23) in the comparator group (*p* = 1.00). Additionally, 71% (*n* = 17/24) rated their experience with the digital health services as effective for improving motivation to exercise regularly with no significant difference when compared to usual care at 48%, (*n* = 11/23, *p* = 0.24). Perceived effectiveness of improving understanding and confidence related to diet and exercise in relation to the digital health services and usual care were also comparable for both groups (Figure 3).

Survey results revealed that since receiving the text messages, 38% (*n* = 9/24) of participants reported changing their diet and 46% (*n* = 11/24) reported changing their exercise habits. Of those participants who received digital health services and completed the survey, 96% (23/24) considered the information provided by the research team on how to use the digital health services to be helpful or very helpful. In the short-answer survey responses, some participants expressed that the continuity and frequency of care received by the digital health services was useful.

However, several participants reported not using the fact sheets (38%, *n* = 8/21) or recipes (24%, *n* = 5/21) of the nutrition app (Figure 2). Similarly, a minority of participants that opted into various digital health components, reported not actually using the videos (18%, *n* = 4/22), text messages (18%, *n* = 4/21), education (23%, *n* = 5/22), or chat feature (32%, *n* = 7/22) of the exercise app.

*Helped to adopt healthier behaviors* was an interview theme that was consistent with perceived effectiveness. Participants described feeling reminded or better informed of healthy behaviors as a result of receiving digital health, particularly the text messages, nutrition apps, and dietetic video consultations. Participants indicated they reflected on the information received before engaging in a particular behavior: “It (text messages) stopped me from adding the wrong things to my meals” (55-years males, kidney transplant). Some participants found the nutrition app effective for trying new recipes and encouraging them to cook: “It gives you different meals and stuff that you don’t eat at home”(55-years male, kidney transplant, intervention group) and “it was saying you get a tomato and you chop it up … you are not just getting up to the microwave and walking back to the television … you are actually doing something because of the what the app (suggested)” (35-years female, kidney transplant, intervention group).

*Reaffirmed what I knew* was another theme that related to perceived effectiveness. Some participants thought it was effective to have information they knew reinforced by the text messages or fact sheets in the nutrition app: “There was a couple of things I read about controlling diabetes … I’ve heard it before but it's nice to be reinforced” (55-years female, CKD).

### Self-efficacy (confidence)

Survey data revealed 77% (*n* = 17/22) participants agreed that they felt capable to do the exercise suggestions from the exercise app unsupervised.

There were three themes related to self-efficacy. The theme *lacking digital competence* highlighted barriers to self-efficacy. Some participants revealed that if they experienced technical issues that they could not resolve themselves, they would stop attending the video consultations or using the app. When asked why they did not reach out to the project officer for assistance, some participants indicated that they felt “lazy” or “embarrassed” to ask. Some participants indicated additional training for setting up digital health would have been beneficial for their engagement: “Would have been useful just to have a bit more information about the setup up (for the video consultations)” (47-years male, CKD).

*Awareness of exercise capabilities* was a theme in part reflecting confidence. For some this related to the amount of physical activity they were able to do based on their health condition/s: “You know it just makes me understand what my body can do more and what pushes my limits” (27-years female, kidney transplant). Some participants indicated that with this knowledge they felt more confident to engage in the exercise more regularly: “It just helps my confidence as well … in getting ready to exercise and exercising more often” (22-years male, liver disease).

*Desire for more tailored and practical approaches* was a theme that indicated unmet values with the digital services. Although many participants felt that the digital health services reaffirmed *and “reinforced”* their knowledge or behaviors, several participants said that they “already knew” the information provided and desired more: “Most of the ones (text messages) I received, I found to be the same ones you get everywhere” (58-years female, kidney transplant). Some participants also perceived that they had diet self-efficacy: “I mean, this might be a bit arrogant (of an) answer, but I think I've got my diet under control. I'm already very careful with what I eat” (65-years male, kidney transplant). Some participants indicated that the information received did not always apply to them: “They used to make me laugh because I get messages that would say, ‘oh, swap out like red meat for lentils three times a week’ and I haven't eaten red meat since I was young” (40-years female, CKD). However, most participants indicated that even after engaging in the study, they wouldn’t have selected the services any differently from what they did when the study commenced. They expressed the need for more tailored and practical *advice*: “I think generally it's quite helpful if it can be more concentrated to us, every person specific needs” (27-years female, kidney transplant) *or “*Yeah it would be nice to give you cooking lessons through the app … or video that you can replay and play through the computer … that would be pretty handy” (64-years male, liver transplant).

### Perceived safety

Seventy-seven percent (*n* = 17/22) of participants who used the exercise app agreed that they felt safe while exercising. Additionally, 38% (*n* = 8/21) of participants agreed or strongly agreed that the nutrition app was as good as seeing a dietitian. Refer to figure 1 for graphical display of these results.

*Professional support to adapt to current health needs* was an interview theme discussed by some participants as contributing to safety. They valued having a health professional to adapt their program to their exercise or dietary needs: “I have a backache … the exercise physiologist would change something (the exercise) to adapt it right away” (55-years female, CKD) or “We had a dietitian we could talk and get an answer … there were a couple of questions (as) we weren’t sure we were doing the right thing” (64-years male, CKD).

## Discussion

This mixed-methods study aimed to evaluate the patients' acceptability of being offered choice from a suite of digital health services to support diet and exercise behaviors in complex chronic conditions by using the TFA. The results found a high acceptability towards several key dimensions for the intervention group, including affective attitudes (seeing the benefit for others and long term use), ethicality (alignment with core values, particularly convenience), minimal burden (ease of use, particularly the exercise app), perceived effectiveness (positive impact on motivation and behavior change), intervention coherence (understanding and valuing that the services aligned with each other), self-efficacy (confidence, particularly with exercising unsupervised), and perceived safety. Lower areas of acceptability included self-efficacy specifically in terms of individualization and digital literacy while opportunity costs associated with competing life demands were a limiting factor. There was no significant difference between participant groups in terms of the quantitatively reported perceived effectiveness in enhancing comprehension, confidence, and motivation.

Our study indicates that heterogeneous patient cohorts with complex clinical needs show varied levels of perceived acceptability for digital health services. Indeed, the diversity of experiences for our study may have been the result of intervention participants selecting different digital health options and having varied levels of engagement with their own choices. However, research shows that ambivalence is typical of digital health interventions in various chronic disease populations.^12^ It suggests there is likely no one size fits all approach; which appears to be a common theme within the literature related to digital health.^47^ Therefore, individualized patient care and flexible delivery options are key factors for effective digital health interventions. Participants highlighted the need for individualized care to enhance their acceptability of the digital health services they used or had access to. The survey results revealed that most participants in the intervention group found the exercise information relevant while less found this to be the case for the dietary information they were provided. This was further supported by the interviews where several participants felt they already knew the information that was provided by the digital health services, particularly in relation to nutrition.

Previous studies have suggested that digital health services for lifestyle behavior management are especially suitable for those recently or newly diagnosed^48,49^ or in an acute recovery phase.^50^ Furthermore, some research also argues that individualizing healthcare goes beyond acceptability research and needs to consider patient-reported psychological and quality-of-life measures.^18,51^ This highlights a need to consider individualizing services based on the various stages of a treatment journey, which might impact participants’ adherence to the proposed interventions and sustainability of behavioral changes.

A high percentage of participants reported in the survey that the information about how to use digital health services was perceived to be effective which was predominantly provided at the beginning of the study. Few also reported in the survey experiencing technical difficulties. However, the interviews revealed varying levels of digital literacy and a real challenge for participants who were less digitally competent. Kidney transplant recipients involved in other digital health lifestyle interventions have valued the information received at the commencement of the study^50^; however, some participants have had concerns with the procedures that followed.^52^ In the current study, some participants admitted to not reaching out for assistance if they had technical issues; and therefore, did not engage with the digital health services they selected. Therefore, actively checking with the user and providing the option for technical support throughout the duration of use should be considered. However, implementing this in clinical practice would be challenging as an additional demand on the workforce. Some research has suggested a digital health support officer role may evolve from this landscape, which has already been identified as of high value among healthcare providers involved in nutrition care.^53^

### Strengths and limitations

In this study the qualitative results provided further depth to the quantitative results and thus it may be considered a more comprehensive assessment. A further strength was surveying both randomized groups (intervention and comparator), to allow for comparisons in perceived effectiveness of the innovative digital services and non-digital usual care services. Using an established framework to guide the analysis helped to structure meaning within the multiple components of acceptability. However, this study had some important limitations. As a result of COVID-19 health pandemic related disruptions, the nondigital usual care comparator group may have been exposed to more telephone services than was usual or expected; therefore, it is not an accurate reflection of usual care within this clinical setting. Furthermore, not surveying, or interviewing participants halfway through the study did not allow comparisons between these time points to identify any differences and/or similarities in these time points could be considered a limitation. Assessment halfway may have captured perspectives of participants more closely to peak engagement which is evidently at the beginning of most mHealth interventions.^54^ Standardized or validated intervention acceptability surveys exist in digital health^55,56^; however, they are highly context-specific and not appropriate for this cohort whereby the research question specifically related to the acceptability of digital health services that were accessed and utilized in this study. The study was conducted in an Australian tertiary hospital context and focused on a specific patient group; and therefore, not generalizable to other countries, or the private sector or relevant to other patient groups.

## Conclusion

This study evaluated the acceptability of patient self-selected digital health diet and exercise services to support people living with complex chronic conditions. The study found that components of these digital health services were perceived to be useful, safe and effective for changing diet and exercise behavior. However, competing life demands, low digital literacy and a desire for more individualized care impacted acceptability of these digital health services. Overall, perceived acceptability of these digital health services varied with this patient group; and therefore, flexibility in care and user choice is important. These findings can assist with the future design and implementation of digital health diet and exercise services in this population.

## Supplemental Material

sj-docx-1-dhj-10.1177_20552076241245278 - Supplemental material for Patients’ acceptability of self-selected digital health services to support diet and exercise among people with complex chronic conditions: Mixed methods study Supplemental material, sj-docx-1-dhj-10.1177_20552076241245278 for Patients’ acceptability of self-selected digital health services to support diet and exercise among people with complex chronic conditions: Mixed methods study by Amandine Barnett, Soraia de Camargo Catapan, Dev K Jegatheesan, Marguerite M Conley, Shelley E Keating, Hannah L Mayr, Lindsey Webb, Riley C C Brown, Jeff S Coombes, Graeme A Macdonald, Nicole M Isbel, Nicola W Burton, Katrina L Campbell, Ingrid J Hickman and Jaimon T Kelly in DIGITAL HEALTH

sj-docx-2-dhj-10.1177_20552076241245278 - Supplemental material for Patients’ acceptability of self-selected digital health services to support diet and exercise among people with complex chronic conditions: Mixed methods study Supplemental material, sj-docx-2-dhj-10.1177_20552076241245278 for Patients’ acceptability of self-selected digital health services to support diet and exercise among people with complex chronic conditions: Mixed methods study by Amandine Barnett, Soraia de Camargo Catapan, Dev K Jegatheesan, Marguerite M Conley, Shelley E Keating, Hannah L Mayr, Lindsey Webb, Riley C C Brown, Jeff S Coombes, Graeme A Macdonald, Nicole M Isbel, Nicola W Burton, Katrina L Campbell, Ingrid J Hickman and Jaimon T Kelly in DIGITAL HEALTH

sj-docx-3-dhj-10.1177_20552076241245278 - Supplemental material for Patients’ acceptability of self-selected digital health services to support diet and exercise among people with complex chronic conditions: Mixed methods study Supplemental material, sj-docx-3-dhj-10.1177_20552076241245278 for Patients’ acceptability of self-selected digital health services to support diet and exercise among people with complex chronic conditions: Mixed methods study by Amandine Barnett, Soraia de Camargo Catapan, Dev K Jegatheesan, Marguerite M Conley, Shelley E Keating, Hannah L Mayr, Lindsey Webb, Riley C C Brown, Jeff S Coombes, Graeme A Macdonald, Nicole M Isbel, Nicola W Burton, Katrina L Campbell, Ingrid J Hickman and Jaimon T Kelly in DIGITAL HEALTH

sj-docx-4-dhj-10.1177_20552076241245278 - Supplemental material for Patients’ acceptability of self-selected digital health services to support diet and exercise among people with complex chronic conditions: Mixed methods study Supplemental material, sj-docx-4-dhj-10.1177_20552076241245278 for Patients’ acceptability of self-selected digital health services to support diet and exercise among people with complex chronic conditions: Mixed methods study by Amandine Barnett, Soraia de Camargo Catapan, Dev K Jegatheesan, Marguerite M Conley, Shelley E Keating, Hannah L Mayr, Lindsey Webb, Riley C C Brown, Jeff S Coombes, Graeme A Macdonald, Nicole M Isbel, Nicola W Burton, Katrina L Campbell, Ingrid J Hickman and Jaimon T Kelly in DIGITAL HEALTH

sj-docx-5-dhj-10.1177_20552076241245278 - Supplemental material for Patients’ acceptability of self-selected digital health services to support diet and exercise among people with complex chronic conditions: Mixed methods study Supplemental material, sj-docx-5-dhj-10.1177_20552076241245278 for Patients’ acceptability of self-selected digital health services to support diet and exercise among people with complex chronic conditions: Mixed methods study by Amandine Barnett, Soraia de Camargo Catapan, Dev K Jegatheesan, Marguerite M Conley, Shelley E Keating, Hannah L Mayr, Lindsey Webb, Riley C C Brown, Jeff S Coombes, Graeme A Macdonald, Nicole M Isbel, Nicola W Burton, Katrina L Campbell, Ingrid J Hickman and Jaimon T Kelly in DIGITAL HEALTH
