# Supplementary material for: Patients’ acceptability of self-selected digital health services to support diet and exercise among people with complex chronic conditions: Mixed methods study
Source: Digit Health. 2024 Jun 7;10:20552076241245278. doi: 10.1177/20552076241245278 (PMC11162125; doi:10.1177/20552076241245278)
Supplement: sj-docx-1-dhj-10.1177_20552076241245278 - Supplemental material for Patients’ acceptability of self-selected digital health services to support diet and exercise among people with complex chronic conditions: Mixed methods study [file sj-docx-1-dhj-10.1177_20552076241245278.docx]

| **Supplementary material 1: Criteria for reporting qualitative studies (COREQ): 32 item checklist** | | |
| --- | --- | --- |
| **No. Item** | **Guide questions/description** | **Reported on page #; section**  **Additional information (if relevant)** |
|  | | |
| **Domain 1: Research team and reflexivity** | | |
| **Personal Characteristics** |  |  |
| 1. Interviewer/facilitator | Which author/s conducted the interview or focus group? | Page 7; Interviews |
| 1. Credentials | What were the researcher’s credentials? E.g. PhD, MD | MNutr&Diet and BEx&NutrSc |
| 1. Occupation | What was their occupation at the time of the study? | PhD candidate and dietitian |
| 1. Gender | Was the researcher male or female? | Female |
| 1. Experience and training | What experience or training did the researcher have? | Page 7; Interviews |
| **Relationship with participants** |  |  |
| 1. Relationship established | Was a relationship established prior to study commencement? | Page 7; Interviews |
| 1. Participant knowledge of the interviewer | What did the participants know about the researcher? e.g. personal goals, reasons for doing the research | Page 6; Participant and Recruitment |
| 1. Interviewer characteristics | What characteristics were reported about the interviewer/facilitator? e.g. Bias, assumptions, reasons and interests in the research topic | Not reported |
|  | | |
| **Domain 2: study design** | | |
| **Theoretical framework** |  |  |
| 1. Methodological orientation and Theory | What methodological orientation was stated to underpin the study? e.g. grounded theory, discourse analysis, ethnography, phenomenology, content analysis | Page 8; Qualitative data |
| **Participant Selection** |  |  |
| 1. Sampling | How were participants selected? e.g. purposive, convenience, consecutive, snowball | Page 5-6; Participants and Recruitment |
| 1. Method of approach | How were participants approached? e.g. face-to-face, telephone, mail, email | Page 6; Participants and Recruitment |
| 1. Sample size | How many participants were in the study? | Page 9; Qualitative data  Page 9; Results |
| 1. Non-participation | How many people refused to participate or dropped out? Reasons? | Page 9; Results |
| Setting |  |  |
| 1. Setting of data collection | Where was the data collected? e.g. home, clinic, workplace | Page 7, Online surveys  Page 7; Interviews |
| 1. Presence of non-participation | Was anyone else present besides the participants and researchers? | Page 7; Interviews |
| 1. Description of sample | What are the important characteristics of the sample? e.g. demographic data, date | Page 5 & 6; Participants & Recruitment |
| Data collection |  |  |
| 1. Interview guide | Were questions, prompts, guides provided by the authors? Was it pilot tested? | Page 7; Interviews |
| 1. Repeat interviews | Were repeat interviews carried out? If yes, how many? | N/A- no repeat interviews were conducted |
| 1. Audio/visual recording | Did the research use audio or visual recording to collect the data? | Page 7; Interviews |
| 1. Field notes | Were field notes made during and/or after the interview or focus group? | No field notes were taken during the interviews |
| 1. Duration | What was the duration of the interviews or focus group? | Page 9; Results |
| 1. Data saturation | Was data saturation discussed? | Page 9; Qualitative data  No- we used information power model instead: |
| 1. Transcripts returned | Were transcripts returned to participants for comment and/or correction? | Transcripts were not returned to participants for comment and/or correction |
|  | | |
| **Domain 3: analysis and findings** | | |
| **Data analysis** |  |  |
| 1. Number of data coders | How many data coders coded the data? | Page 8; Qualitative data  Two |
| 1. Description of coding tree | Did authors provide a description of the coding tree? | No description of coding tree was provided. |
| 1. Derivation of themes | Were themes identified in advance or derived from the data? | Page 8; Qualitative data  Derived from the Data |
| 1. Software | What software, if applicable, was used to manage the data? | Page 8; Qualitative data |
| 1. Participant checking | Did participants provide feedback on the findings? | No |
| Reporting |  |  |
| 1. Quotations presented | Were participant quotations presented to illustrate the themes / findings? Was each quotation identified? e.g. participant number | Page 10 to 16; Results |
| 1. Data and findings consistent | Was there consistency between the data presented and the findings? | Pages 17 to 18; Discussion |
| 1. Clarity of major themes | Were major themes clearly presented in the findings? | Pages 10 to 17 Results |
| 1. Clarity of minor themes | Is there a description of diverse cases or discussion of minor themes? | Pages 10 to 17 Results  And Table 2 |
