# Supplementary material for: Patients’ acceptability of self-selected digital health services to support diet and exercise among people with complex chronic conditions: Mixed methods study
Source: Digit Health. 2024 Jun 7;10:20552076241245278. doi: 10.1177/20552076241245278 (PMC11162125; doi:10.1177/20552076241245278)
Supplement: sj-docx-3-dhj-10.1177_20552076241245278 - Supplemental material for Patients’ acceptability of self-selected digital health services to support diet and exercise among people with complex chronic conditions: Mixed methods study [file sj-docx-3-dhj-10.1177_20552076241245278.docx]

**Supplementary material 3: Good Reporting of A Mixed Methods Study (GRAMMS) checklist**

| **GRAMMS Criteria** | **Page number; section** |
| --- | --- |
| (1) Describe the justification for using a mixed methods approach to the research question | Page 4- Study design and setting |
| (2) Describe the design in terms of the purpose, priority, and sequence of methods | Page 6 to 7; Data collection  Page 7 to 9; Data analysis |
| (3) Describe each method in terms of sampling, data collection and analysis | Page 5 to 6; Participant Recruitment  Page 6 to 7; Data collection  Page 7 to 9; Data analysis |
| (4) Describe where integration has occurred, how it has occurred and who has participated in it | Page 9; Data Analysis (Integration and presentation of results) |
| (5) Describe any limitation of one method associated with the present of the other method | Page 19; Discussion (Strengths and limitations) |
| (6) Describe any insights gained from mixing or integrating methods | Page 17-20; Discussion |
