# Supplementary material for: Patients’ acceptability of self-selected digital health services to support diet and exercise among people with complex chronic conditions: Mixed methods study
Source: Digit Health. 2024 Jun 7;10:20552076241245278. doi: 10.1177/20552076241245278 (PMC11162125; doi:10.1177/20552076241245278)
Supplement: sj-docx-4-dhj-10.1177_20552076241245278 - Supplemental material for Patients’ acceptability of self-selected digital health services to support diet and exercise among people with complex chronic conditions: Mixed methods study [file sj-docx-4-dhj-10.1177_20552076241245278.docx]

| **Supplementary material 4: Survey and Interview questionnaires used in this study**  **Table S4-1: Survey questionnaire** | | | | | | | | | | | | |
| --- | --- | --- | --- | --- | --- | --- | --- | --- | --- | --- | --- | --- |
| **Questions and Items** | **Response type** | | | | | | | | | | | |
| **Overall** | | | | | | | | | | | | |
| Overall, how would you rate the support you received from the service to improve diet and exercise? | Very satisfied | Satisfied | | | Neutral | | | Unsatisfied | | Very unsatisfied | | |
| How would you rate the information provided by the research team on how to use the technology in this study? | Very helpful | Helpful | | | Neither | | | Unhelpful | | Very unhelpful | | |
| **Session with dietitian (Comparator group)** |  | | | | | | | | | | | |
| Please fill in the blank with the options provided  The dietitian was _______________ at improving my understanding about the importance of diet | Very effective | Effective | | | Average | | | Ineffective | | Very ineffective | | |
| Please fill in the blank with the options provided  The dietitian was _______________ at improving my understanding about the importance of exercise | Very effective | Effective | | | Average | | | Ineffective | | Very ineffective | | |
| Please fill in the blank with the options provided  The dietitian was _______________ at improving my confidence to choose healthy meal and snack options for myself | Very effective | Effective | | | Average | | | Ineffective | | Very ineffective | | |
| Please fill in the blank with the options provided  The dietitian was _______________ at improving my confidence to choose suitable exercise options for myself | Very effective | Effective | | | Average | | | Ineffective | | Very ineffective | | |
| Please fill in the blank with the options provided  The dietitian was _______________ at improving my motivation to eat healthily | Very effective | Effective | | | Average | | | Ineffective | | Very ineffective | | |
| Please fill in the blank with the options provided  The dietitian was _______________ at improving my motivation to exercise regularly | Very effective | Effective | | | Average | | | Ineffective | | Very ineffective | | |
| The information the dietitian provided about diet was relevant to me | Strongly agree | Agree | | | No opinion or uncertain | | | disagree | | Strongly disagree | | |
| The information the dietitian provided about exercise was relevant to me | Strongly agree | Agree | | | No opinion or uncertain | | | disagree | | Strongly disagree | | |
| **Overall digital health use (Intervention group)** |  | | | | | | | | | | | |
| This digital health program is something I could see being offered long term to other outpatients at the hospital | Strongly agree | Agree | | | No opinion or uncertain | | | disagree | | Strongly disagree | | |
| Please fill in the blank with the options provided  The technology options were _______________ at improving my understanding about the importance of diet | Very effective | Effective | | | Average | | | Ineffective | | Very ineffective | | |
| Please fill in the blank with the options provided  The technology options were _______________ at improving my understanding about the importance of exercise | Very effective | Effective | | | Average | | | Ineffective | | Very ineffective | | |
| Please fill in the blank with the options provided  The technology options were _______________ at improving my confidence to choose healthy meal and snack options for myself | Very effective | Effective | | | Average | | | Ineffective | | Very ineffective | | |
| Please fill in the blank with the options provided  The technology options were _______________ at improving my confidence to choose suitable exercise options for myself | Very effective | Effective | | | Average | | | Ineffective | | Very ineffective | | |
| Please fill in the blank with the options provided  The technology options were _______________ at improving my motivation to eat healthily | Very effective | Effective | | | Average | | | Ineffective | | Very ineffective | | |
| Please fill in the blank with the options provided  The technology options were _______________ at improving my motivation to exercise regularly | Very effective | Effective | | | Average | | | Ineffective | | Very ineffective | | |
| The information provided via the technology about diet was relevant to me | Strongly agree | Agree | | | No opinion or uncertain | | | disagree | | Strongly disagree | | |
| The information provided via the technology about exercise was relevant to me | Strongly agree | Agree | | | No opinion or uncertain | | | disagree | | Strongly disagree | | |
| Have you shared information from any of the technology platforms with others? | Provide options: Yes or No  Option to write who if answered yes | | | | | | | | | | | |
| Did you encounter any technical issues with any of the technology during the study? | Almost Always | Frequently | | | Sometimes | | | Occasionally | | Never | | |
| **Questions specifically relating to text messages** |  | | | | | | | | | | | |
| How often did you read the text message/s? | Almost Always | Frequently | | | Sometimes | | | Occasionally | | Never | | |
| Was the time of day that the text message/s were sent suitable for you? | Very good | Good | | | No opinion | | | Not good | | Not very good | | |
| Since receiving the diet text messages, did you make any changes to where you eat your meals and snacks? | Yes | | | | | | No | | | | | |
| What changes did you make in relation to diet text messages? (You may select more than one) | Provide options (can select multiple): Yes, I’m not longer eating at my desk; Yes, I’m not sitting in front of the tv/devices; Yes, I’m eating with others; No, I haven’t made any changes, other [option to write] | | | | | | | | | | | |
| Since receiving the exercise text messages, did you make any changes in where you exercise? | Yes | | | | | | No | | | | | |
| What changes did you make in relation to exercise text messages? (You may select more than one) | Provide options: Yes, I’ve tried exercising at home; Yes, I’ve tried exercising outside; No, I haven’t made any changes to exercise; other [ option to write] | | | | | | | | | | | |
| Since receiving the exercise text messages, have you exercised more with family and friends? | Almost Always | | Frequently | | | Sometimes | | | Occasionally | Never | | |
| **Questions relating to the Nutrition app** | | | | | | | | | | | | |
| How did you use the nutrition app or website? | Via the App | | | Via the Website | | | I used both | | | I did not use | | |
| How would you describe your experience using each of the following features of the Sophus nutrition app/website?  1.Fact Sheets ;2. Educational videos; 3. Recipes; 4. Reflective journal | For each component rate across five-level Likert Scale (Very easy, easy, Neither easy, nor difficult, difficult, very difficult)  Option: did not use | | | | | | | | | | | |
| Would you recommend the nutrition app/website to others with your health condition? | Strongly agree | Agree | | | No opinion or uncertain | | | disagree | | Strongly disagree | | Did not use |
| Do you feel that the information in the nutrition app/ website is as good as seeing a dietitian? | Strongly agree | Agree | | | No opinion or uncertain | | | disagree | | Strongly disagree | | Did not use |
| **Questions relating to exercise app** |  | | | | | | | | | | | |
| Did you feel capable to do the exercise suggestions from the exercise app at home or outside? | Strongly agree | Agree | | | No opinion or uncertain | | | disagree | | Strongly disagree | | Did not use |
| How would you describe your experience using each of the following features of the exercise app?  1.Video demonstrations of exercises; 2. Text-based instructions of exercises; 3. Educational content; 4. Built in chat features with an AEP | For each component rate across five-level Likert Scale (Very easy, easy, Neither easy, nor difficult, difficult, very difficult)  Option: did not use | | | | | | | | | | | |
| Did you feel safe while exercising based on the information from the exercise app? | Strongly agree | Agree | | | No opinion or uncertain | | | disagree | | Strongly disagree | | Did not use |
| Would you recommend the exercise app to others with your health condition? | Strongly agree | Agree | | | No opinion or uncertain | | | disagree | | Strongly disagree | Did not use | |
| **Questions relating to diet video consult (INTERVENTION GROUP AND ONLY IF SELECTED AS AN OPTION)** | | | | | | | | | | | | |
| How satisfied were you with the diet video consult? | Very Satisfied | Satisfied | | | Neutral | | | Unsatisfied | | Very unsatisfied | Did not use | |
| **Questions relating to exercise video consult (INTERVENTION GROUP AND ONLY IF SELECTED AS AN OPTION)** | | | | | | | | | | | | |
| How satisfied were you with the exercise video consult? | Very Satisfied | Satisfied | | | Neutral | | | Unsatisfied | | Very Unsatisfied | Did not use | |

**Table S4-2: Interview questionnaire**

| 1) There were several different text messages that you received (show/state examples – one education – one social – one goal setting). Could you tell me whether the text messages had an impact on your behavior in relation to diet and why? |
| --- |
| 2) Could you tell me whether the text messages had an impact on your behavior in relation to exercise and why? |
| 3) How have you used the nutrition and exercise app? |
| 4) Do you think these app/s could improve in any way? If so, what could change? |
| 5) Could you tell me about your impressions of using the nutrition app? |
| 6) Can you tell me about your impressions of using the exercise app? |
| 7) Can you tell me your impressions of attending the diet video consult? |
| 8) Can you tell me your impressions of attending the exercise video consult? |
| 9) How do you think the technology can be improved? |
| 10) Now that you have engaged with the study, would have chosen any different technology to what you had chosen? |
| 11) Were there any further comments you would like to make regarding the study? |
