# Supplementary material for: Patients’ acceptability of self-selected digital health services to support diet and exercise among people with complex chronic conditions: Mixed methods study
Source: Digit Health. 2024 Jun 7;10:20552076241245278. doi: 10.1177/20552076241245278 (PMC11162125; doi:10.1177/20552076241245278)
Supplement: sj-docx-5-dhj-10.1177_20552076241245278 - Supplemental material for Patients’ acceptability of self-selected digital health services to support diet and exercise among people with complex chronic conditions: Mixed methods study [file sj-docx-5-dhj-10.1177_20552076241245278.docx]

**Supplementary material 5: Survey Results**

| **Table S5-1: Overall Satisfaction with support received** | | | | | |
| --- | --- | --- | --- | --- | --- |
| Survey item | Very satisfied  n (%) | Satisfied  n (%) | Neutral  n (%) | Unsatisfied  n (%) | Very unsatisfied  n (%) |
| Overall, how would you rate the support you received from the service to improve diet and exercise?  Comparator: n=26  Intervention: n= 24 | Comparator:  5 (19.2) | Comparato:  10 (38.5) | Comparator:  7(26.9) | Comparator:  3(11.5) | Comparator:  1(3.8) |
|  | Intervention  10 (41.7) | Intervention:  10 (41.7) | Intervention:  3 (12.5) | Interventio:0 (0) | Intervention:  1(4.2) |

| **Table S5-2: Whether information provided about technology was helpful** | | | | | |
| --- | --- | --- | --- | --- | --- |
| Survey item | Very Helpful  n (%) | Helpful  n (%) | Neither  n (%) | Unhelpful  n (%) | Very unhelpful  n (%) |
| How would you rate the information provided by the research team on how to use the technology in this study?(n= 24) | Intervention:  11(45.8) | Intervention:  12(50) | Intervention:  0 (0) | Intervention:  1(4.2) | Intervention:  0(0) |

**Digital health monitoring**

| **Table S5-3: Effectiveness of dietitian appointments or digital health service options** | | | | | |
| --- | --- | --- | --- | --- | --- |
| Survey item | Very effective  n (%) | Effective  n (%) | Average  n (%) | Ineffective  n (%) | Very ineffective  n (%) |
| The dietitian was _______________  at improving my **understanding**  about the importance of diet (n=23) | 5 (21.7) | 10 (43.5) | 6 (26.1) | 1 (4.3) | 1 (4.3) |
| The technology options were  _______________ at improving my  **understanding** about the  importance of diet (n=24) | 4 (16.7) | 11 (45.8) | 6 (25.0) | 3 (12.5) | 0 (0) |
|  | | | | | |
| The dietitian was _______________  at improving my **understanding**  about the importance of exercise (n=23) | 4 (17.3) | 9 (39.1) | 6 (26.1) | 3 (13.0) | 1 (4.3) |
| The technology options were  _______________ at improving my  **understanding** about the  importance of exercise  (n=24) | 6 (25.0) | 14 (58.3) | 3 (12.5) | 1 (4.2) | 0 (0) |
|  | | | | | |
| The dietitian was _______________  at improving my **confidence** to  choose healthy meal and snack  options for myself (n=23) | 5 (21.7) | 9 (39.1) | 7 (30.4) | 1 (4.3) | 1 (4.3) |
| The technology options were  _______________ at improving my  **confidence** to choose healthy  meal and snack options for  myself (n=24 | 2 (8.3) | 12 (50.0) | 7 (29.2) | 3 (12.5) | 0 (0) |
|  | | | | | |
| The dietitian was _______________  at improving my **confidence** to  choose suitable exercise options  for myself (n=23) | 3 (13.0) | 9 (39.1) | 7 (30.4) | 2 (8.7) | 2 (8.7) |
| The technology options were  _______________ at improving my  **confidence** to choose suitable  exercise options for myself (n=24) | 4 (16.7) | 16 (66.7) | 4 (16.7) | 0 (0) | 0 (0) |
|  | | | | | |
| The dietitian was _______________  at improving my **motivation** to  eat healthily  (n=23) | 4 (17.4) | 10 (43.5) | 5 (21.7) | 3 (13.0) | 1 (4.3) |
| The technology options were  _______________ at improving my  **motivation** to eat healthily  (n=24) | 1 (4.2) | 15 (62.5) | 5 (20.8) | 2 (8.3) | 1 (4.2) |
|  | | | | | |
| The dietitian was _______________  at improving my **motivation** to  exercise regularly (n=23) | 3 (13.0) | 8 (34.8) | 7 (30.4) | 3 (13.0) | 2 (8.7) |
| The technology options were  _______________ at improving my  **motivation** to exercise regularly (n=24) | 6 (25.0) | 11 (45.8) | 4 (16.7) | 3 (12.5) | 0 (0) |

| **Table S5-4: Relevancy of information provided in the dietitian appointments or digital health service options** | | | | | |
| --- | --- | --- | --- | --- | --- |
| Survey item | Strongly agree n (%) | Agree  n (%) | No opinion or uncertain  n (%) | Disagree  n (%) | Strongly disagree  n (%) |
| The information the dietitian provided about diet was relevant (n=23) | 4 (17.4) | 14 (60.9) | 4 (17.4) | 0 (0) | 1 (4.3) |
| The information provided via the technology about diet was relevant to me (n=24) | 4 (16.7) | 10 (41.7) | 7 (29.2) | 3 (12.5) | 0 (0) |
|  | | | | | |
| The Information the dietitian provided about exercise was relevant to me  (n-22) | 3 (14) | 9 (41) | 7 (32) | 2 (9) | 1(4) |
| The information provided via the technology about exercise was relevant to me (n=24) | 7 (29.2) | 14 (58.3) | 2 (8.3) | 1 (4.2) | 0 (0) |

| **Table S5-5: Sharing of information provided in the dietitian appointments or digital health services** | | |
| --- | --- | --- |
| Survey item | Yes  n (%) | No  n (%) |
| Have you shared information from your session with the  dietitian with others? (n=23) | 9 (39.1) | 14 (60.9) |
| Have you shared information from any of the technology platforms with others? (n=24) | 9 (37.5) | 15 (62.5) |

| **Table S5-6: Perceptions on whether it could be offered long term** | | | | | |
| --- | --- | --- | --- | --- | --- |
| Survey item | Strongly agree  n (%) | Agree  n (%) | No opinion or uncertain n( %) | Disagree  n (%) | Strongly disagree  n (%) |
| This digital health service program is something I could see being offered long term to other outpatients at the hospital (n=24) | 7 (29.2) | 13 (54.2) | 3 (12.5) | 1 (4.2) | 0 (0) |

**Perceptions and experiences with technology in the intervention group**

| **Table S5-7: Technical issues encountered** | | | | | |
| --- | --- | --- | --- | --- | --- |
| Survey item | Almost always  n (%) | Frequently  n (%) | Sometimes  n (%) | Occasionally  n (%) | Never  n (%) |
| Did you encounter any technical issues with any of the technology during the study? (n=24)* | 1 (4.2) | 4(16.7) | 4(16.7) | 7(29.2) | 8 (33.3) |

*This refers to both the digital health services as well as assessment and monitoring tools.

**Text messages**

| **Table S5-8: Frequency with reading text messages and whether exercising more with family and friends** | | | | | |
| --- | --- | --- | --- | --- | --- |
| Survey item | Almost always  n (%) | Frequently  n (%) | Sometimes  n (%) | Occasionally  n (%) | Never  n (%) |
| How often did you read the text  message/s? (n=24) | 12 (50.0) | 5 (20.8) | 4 (16.7) | 3 (12.5) | 0 (0) |
| Since receiving the exercise text  messages, have you exercised  more with family and friends? (n-24) | 0 (0) | 2 (8.3) | 10 (41.7) | 5 (20.8) | 7 (29.2) |

| **Table S5-9: Suitability of time text messages were sent** | | | | | |
| --- | --- | --- | --- | --- | --- |
| Survey item | Very good  n (%) | Good  n (%) | No opinion  n (%) | Not good  n (%) | Not very good  n (%) |
| Was the time of day that the text message/s were sent suitable for you? (n=24) | 5 (20.8) | 14 (58.3) | 4 (16.7) | 1 (4.2) | 0 (0) |

| **Table S5-10: Changes made as result of the diet text messages** | | | | |
| --- | --- | --- | --- | --- |
| Survey item | Yes- I’m no longer eating at my desk  n (%) | Yes- I’m longer sitting in front of the tv/devices  n (%) | Yes- I’m eating with others  n (%) | Yes- other  n (%) |
| What changes did you make in relation to diet text messages? (You may select more than one) (n=24) | 2 (8.3) | 2(8.3) | 6 (25.0) | 1 (4.2) |

| **Table S5-11: Changes as result of text messages** | | |
| --- | --- | --- |
| Survey item | Yes  n (%) | No  n (%) |
| Since receiving the diet text messages, did you make any changes to where you eat your meals and snacks? (n=24) | 9 (37.5) | 15 (62.5) |
| Since receiving the exercise text messages, did you make any changes to where you exercise? (n=24) | 11 (45.8) | 13 (54.2) |

| **Table S5-12: Changes made as result of the exercise text messages** | | | |
| --- | --- | --- | --- |
| Survey item | I’ve exercised at home  n (%) | I’ve exercised outside  n (%) | Other  n (%) |
| What changes did you make in relation to exercise text messages? (You may select more than one) (n=24) | 6 (25.0) | 6 (25.0) | 1 (4.2) |

**Nutrition app**

| **Table S5-13: Changes made as result of the text message** | | | | |
| --- | --- | --- | --- | --- |
| Survey item | Via the app  n (%) | Via the website  n (%) | I used both  n (%) | I did not use it at all  n (%) |
| How did you use the Sophus Nutrition app or website? (n=21) | 9 (42.9) | 1 (4.8) | 3 (14.3) | 8 (38.1) |

| **Table S5-14: Experience with features of the nutrition app** | | | | | | |
| --- | --- | --- | --- | --- | --- | --- |
| Survey item | Very easy  n (%) | Easy  n (%) | Neither  n (%) | Difficult  n (%) | Very difficult  n (%) | Did not use  n (%) |
| Fact sheets (n=21) | 3 (14.3) | 4 (19.0) | 6 (28.6) | 0 (0) | 0 (0) | 8 (38.1) |
| Educational videos (n=21) | 2 (9.5) | 5 (23.8) | 5 (23.8) | 1 (4.8) | 0 (0) | 8 (38.1) |
| Recipes (n=21) | 3 (14.3) | 5 (23.8) | 7 (33.3) | 1 (4.8) | 0 (0) | 5 (23.8) |

| **Table S5-15: Suitability of the nutrition app** | | | | | | |
| --- | --- | --- | --- | --- | --- | --- |
| Survey item | Strongly agree.  n (%) | Agree  n (%) | No opinion or uncertain  n (%) | Disagree  n (%) | Strongly disagree  n (%) | Did not use  n (%) |
| Would you recommend the  Nutrition app to others  with your health condition? (n=21) | 5 (23.8) | 5 (23.8) | 4 (19.0) | 1 (4.8) | 1 (4.8) | 5 (23.8) * |
| Do you feel that the information?  in the nutrition app is as  good as seeing a dietitian? (n=21) | 3 (14.3) | 5 (23.8) | 5 (23.8) | 3 (14.3) | 1 (4.8) | 4 (19.0) |

*One participant reported using it for this first survey item regarding the suitability of the nutrition app but not the second item

**Exercise app**

| **Table S5-16: Experience with features of the exercise app** | | | | | | |
| --- | --- | --- | --- | --- | --- | --- |
| Survey item | Very easy  n (%) | Easy  n (%) | Neither  n (%) | Difficult  n (%) | Very difficult  n (%) | Did not use  n (%) |
| Video demonstrations with exercises (n=22) | 9 (40.9) | 7 (31.8) | 2 (9.1) | 0 (0) | 0 (0) | 4 (18.2) |
| Text-based instructions of exercises (n=22) | 7 (31.8) | 7 (31.8) | 4 (18.2) | 0 (0) | 0 (0) | 4 (18.2) |
| Educational content (n=22) | 5 (22.7) | 9 (40.9) | 2 (9.1) | 1 (4.5) | 0 (0) | 5 (22.7) |
| Built in chat features with the exercise physiologist (n=22) | 3 (13.64) | 6 (27.3) | 6 (27.3) | 0 (0) | 0 (0) | 7 (31.8) |

| **Table S5-17: Suitability of the exercise app** | | | | | | |
| --- | --- | --- | --- | --- | --- | --- |
| Survey item | Strongly agree  n (%) | Agree  n (%) | No opinion or uncertain  n (%) | Disagree  n (%) | Strongly disagree  n (%) | Did not use  n (%) |
| Did you feel capable to do the exercise suggestions from the exercise app at home or outside?  (n=22) | 8 (36.4) | 6 (27.3) | 4 (18.2) | 1 (4.5) | 0 (0) | 3 (13.6) |
| Did you feel safe while exercising based on the information from the exercise app? (n=22) | 8 (36.4) | 9 (40.9) | 2 (9.1) | 0 (0) | 0 (0) | 3 (13.6) |
| Would you recommend the  exercise app to others with your  health condition?  (n=22) | 7 (31.8) | 7(31.8) | 5 (22.7) | 0 (0) | 0 (0) | 3 (13.6) |

**Digital health group sessions**

| **Table S5-18: Satisfaction with the exercise app** | | | | | | |
| --- | --- | --- | --- | --- | --- | --- |
| Survey item | Very satisfied  n (%) | Satisfied  n (%) | Neutral  n (%) | Unsatisfied  n (%) | Very unsatisfied  n (%) | Did not use  n (%) |
| How satisfied were you with the diet video consult? (n=20) | 5 (25.0) | 6 (30.0) | 4 (20.0) | 1 (5.0) | 0 (0) | 4 (20.0) |
| How satisfied were you with the  exercise video consult? (n=20) | 10 (50.0) | 4 (20.0) | 2 (10.0) | 2 (10.0) | 2 (10.0) | 0 (0.0) |
